# 1169. Derivation And Validation of an International Clinical Prognostication Model for 28-day Sepsis Mortality.

**DOI:** 10.1093/ofid/ofac492.1006

**Published:** 2022-12-15

**Authors:** Paul W Blair, Rittal Mehta, Tin Som, Stephen Okello, Ephraim L Tsalik, Abdullah Wailagala, Christopher W Woods, Michael Prouty, Josh Chenoweth, Andrew Letizia, Dennis Faix, Nehkonti Adams, Emily R Ko, Chris Duplessis, Mohammed Lamorde, Alex Owusu-Ofori, Prossy Naluyima, Mubaraka Kayiira, Chris Oppong, Michelle Rozo, Ann Fox, James Lawler, Peter Waitt, Angela Prouty, Te Vantha, Charmagne Beckett, Hannah Kibuuka, George Oduro, Kevin Schully, Danielle Clark, Danielle Clark, Danielle Clark

**Affiliations:** Austere environments Consortium for Enhanced Sepsis Outcomes (ACESO), Henry M. Jackson Foundation for the Advancement of Military Medicine, Bethesda, Maryland; Henry M. Jackson Foundation, Bethesda, Maryland; Takeo Provincial Referral Hospital, Takeo, Takeo, Cambodia; Makerere University Walter Reed Project, Kampala, Kampala, Uganda; Duke University Division of Infectious Diseases, Duke University School of Medicine, Durham, Durham, North Carolina; Infectious Diseases Institute, Kampala, Kampala, Uganda; Duke University Medical Center, Durham, North Carolina; Naval Medical Research Unit-2, Phnom Penh, Phnom Penh, Cambodia; Henry M. Jackson Foundation, Bethesda, Maryland; Naval Medical Research Unit-3 Ghana Detachment, Accra, Greater Accra, Ghana; Naval Medical Research Unit-2, Phnom Penh, Phnom Penh, Cambodia; Naval Medical Research Center Infectious Diseases Directorate, Bethesda, Maryland; Duke University School of Medicine, Durham, Durham, NC; Naval Medical Research Center Infectious Diseases Directorate, Bethesda, Maryland; Infectious Diseases Institute, Kampala, Kampala, Uganda; Komfo Anokye Teaching Hospital,, kumasi, Central, Ghana; Makerere University Walter Reed Project, Kampala, Kampala, Uganda; Infectious Diseases Institute, Kampala, Kampala, Kampala, Uganda; Komfo Anokye Teaching Hospital, bethesda, Maryland; Austere environments Consortium for Enhanced Sepsis Outcomes (ACESO), Henry M. Jackson Foundation for the Advancement of Military Medicine, Bethesda, Maryland; Naval Medical Research Unit-3 Ghana Detachment, Accra, Greater Accra, Ghana; Global Center for Health Security at Nebraska and Division of Infectious Disease, Department of Internal Medicine, Omaha, Nebraska; Infectious Diseases Institute, Kampala, Kampala, Uganda; Naval Medical Research Unit-2, Phnom Penh, Phnom Penh, Cambodia; Takeo Provincial Referral Hospital, Takeo, Takeo, Cambodia; Naval Medical Research Center Infectious Diseases Directorate, Bethesda, Maryland; Makerere University Walter Reed Project, Kampala, Kampala, Uganda; Komfo Anokye Teaching Hospital, bethesda, Maryland; Naval Medical Research Center Infectious Diseases Directorate, Bethesda, Maryland; The Henry M. Jackson Foundation for the Advancement of Military Medicine, Inc., Bethesda, MD, Bethesda, Maryland; The Henry M. Jackson Foundation for the Advancement of Military Medicine, Inc., Bethesda, MD, Bethesda, Maryland; The Henry M. Jackson Foundation for the Advancement of Military Medicine, Inc., Bethesda, MD, Bethesda, Maryland

## Abstract

**Background:**

Survival prediction models have largely been derived and validated only in high-resource Western countries or in single center studies. We sought to create a prediction model for 28-day mortality using laboratory and physiologic parameters from 3 international sepsis cohorts and externally validated the model.

**Methods:**

During 2014 to 2021, adult hospitalized patients with suspected infection were enrolled in Durham, United States (N=180) and those with suspected infection and ≥2 SIRS (Systemic Inflammatory Response Syndrome) criteria in Takeo, Cambodia (N=200), and Kumasi, Ghana (N=187). Twenty-five clinical laboratory and physiologic parameters were candidate covariates and sepsis screening scores included as comparators. First, bivariate Cox regression models were performed to determine risk of individual parameters. Then, a 10-fold cross-validated forward stepwise model selection technique was used to eliminate nonsignificant variables using a p-value < 0.10 and the cross-validated C-statistic was estimated. Lastly, this model was applied to an external cohort of hospitalized adults with suspected infection and ≥2 SIRS in Fort Portal, Uganda (N=331 with 9.3% 28-day mortality).

**Results:**

Among 567 participants, overall mortality was 16.4% at 28-days. Mortality rate highest in Ghana (31.0%), followed by Cambodia (11.0%) and the United States (7.2%). Bivariate analyses identified hypernatremia ( >145 mEq/L) being associated with the highest risk of death (hazard ratio: 7.42; 95% CI: 3.65 to 15.10; Figure 1). On multivariable analysis, a 28-day mortality model including mean arterial pressure, Glasgow Coma score, blood sodium, lactate, and blood urea nitrogen (Table 1) resulted in a 10-fold cross-validated C-statistic of 0.80 (95% CI: 0.61 to 0.88). This model predicted mortality accurately in the validation cohort with a C-statistic of 0.74 (95%CI: 0.69 to 0.79).

**Figure:**
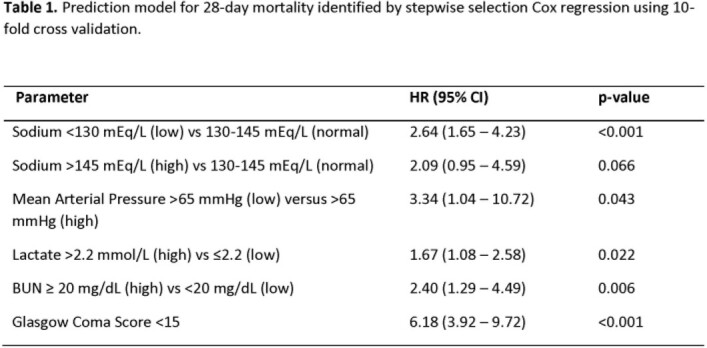

**Figure 1.**
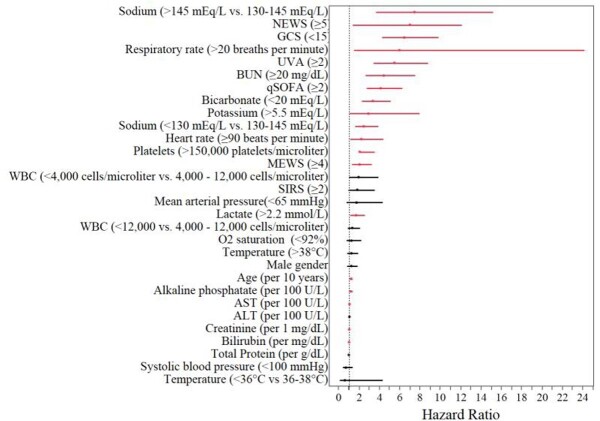
Forest plot for bivariate analyses for one month survival across United States, Cambodia, and Ghana cohorts.

**Conclusion:**

Hypotension, altered mental status, serum sodium, serum BUN, and plasma lactate accurately identified risk of death by 28-days among those with suspected sepsis in 3 international derivation cohorts and in a validation cohort in Uganda. Our findings emphasize the importance of clinical laboratory results for sepsis risk stratification.

**Disclosures:**

**Ephraim L. Tsalik, MD PhD**, Danaher Diagnostics, Predigen, and Biomeme: In the past 3 years, I have had held equity and consulted for Predigen and Biomeme. Currently, I am an employee of Danaher Diagnostics. **Christopher W. Woods, MD MPH**, Predigen, Inc: Co-founder.

